# Nanoelectronics: Materials, Devices, and Applications (Second Edition)

**DOI:** 10.3390/nano15211636

**Published:** 2025-10-27

**Authors:** Chunchang Wang

**Affiliations:** Laboratory of Dielectric Functional Materials, School of Materials Science & Engineering, Anhui University, Hefei 230601, China; ccwang@ahu.edu.cn

The field of nanoelectronics continues to be a cornerstone of modern technological advancement, pushing the boundaries of what is possible in computing, sensing, and energy technologies. This Special Issue, “Nanoelectronics: Materials, Devices and Applications (Second Edition),” serves as a testament to the vibrant and interdisciplinary research efforts dedicated to understanding and harnessing the unique properties of matter at the nanoscale. The diverse collection of articles within this Special Issue highlights significant progress in the synthesis of novel materials, the innovative design of devices, and the exploration of their applications across a wide spectrum.

A prominent theme in this Special Issue is the relentless pursuit of alternative and sustainable energy sources. The work by Zhao et al. [1] introduces a novel water evaporation-driven nanogenerator (S-WEG) based on the natural mineral sepiolite. This research elegantly demonstrates how the hierarchical nanoporous structure and intrinsic hydrophilicity of a nanomaterial can be leveraged to harvest energy from ambient humidity, offering a promising pathway for powering small-scale electronics in an eco-friendly manner.

Equally important is the development of novel materials with exceptional physical properties. Lv et al. [2] present a gadolinium(III)-based metal–organic framework (MOF) exhibiting a significant magnetocaloric effect. This material shows great potential as a highly efficient magnetic refrigerant for cryogenic applications, positioning it as a viable candidate to replace scarce helium-3. On another front, Kim and Choi [3] explore composite materials, demonstrating that a stacked nanocomposite active layer of zinc–tin oxide and single-walled carbon nanotubes (ZTO/SWNTs) can significantly enhance the performance of solution-processed thin-film transistors (TFTs). This approach offers a promising route for the low-cost fabrication of high-performance flexible electronics.

A significant portion of the research focuses on the critical area of memory and neuromorphic computing devices, which are essential for the next generation of data processing. Jang et al. [4] employ an innovative laser-driven technique to induce permanent, localized strain in molybdenum disulfide (MoS_2_), resulting in resistive switching memory (ReRAM) devices with a 30% reduction in operating voltage and improved endurance. Complementing this, Kim and Lee [5] utilize advanced Technology Computer-Aided Design (TCAD) simulations to model ReRAM devices based on HfO_2_-Al_2_O_3_ stacks. Their work provides crucial insights into how device structure influences conductance behavior, directly linking these characteristics to the accuracy of neuromorphic computing systems performing tasks like image recognition.

Furthermore, this Special Issue addresses the fundamental impact of fabrication processes on material properties. Majkowycz et al. [6] present a meticulous comparative study on the influence of wet chemical versus dry reactive ion etching (RIE) on the defect levels in two key infrared detector materials: InAs/InAsSb type-II superlattices and mercury cadmium telluride (MCT). Their findings, obtained through deep-level transient spectroscopy (DLTS), are invaluable for optimizing fabrication processes to minimize performance-degrading defects.

Finally, this Special Issue ventures into the cutting-edge realms of quantum and photonic applications. Gao et al. [7] theoretically investigate the Josephson diode effect (JDE) in a complex system involving double quantum dots coupled to unalike Majorana nanowires. Their work reveals how quantum interference can lead to a non-reciprocal supercurrent, a phenomenon with profound implications for the development of superconducting quantum electronics. In the photonic domain, Chen et al. [8] demonstrate a GdFe-based nanocavity-shaped metasurface capable of efficient infrared light absorption and manipulation through the excitation and resonant accumulation of spatial electromagnetic wavefields, opening new avenues for IR photonics and sensing.

This collection is aptly concluded by the comprehensive review by Liu and Liu [9] on flexible sensors for wearable devices. This review not only summarizes the tremendous progress in this applied field but also thoughtfully outlines the ongoing challenges of miniaturization, integration, sustainability, and energy efficiency, effectively charting the course for future research.

The breadth and depth of the research presented in this Special Issue underscore a collective movement from fundamental material exploration to sophisticated device engineering and practical application. We extend our sincere gratitude to all the authors and reviewers who have contributed their expertise to this project. It is our firm belief that the insights and discoveries contained within these pages will inspire further innovation and accelerate the development of nanoelectronics, ultimately shaping the future of technology.
